# The Most Efficacious Form of Arsenic for Devitalizing Dental Pulps

**Published:** 1874-08

**Authors:** Thomas Burgh


### ARTICLE VI.

### *The Most Efficacious Form of Arsenic for Devitalizing Dental Pulps.*

BY THOMAS BURGH, D. D. S.

All who have used arsenic for this purpose, and who seek to thoroughly extirpate the pulp, have probably found some difficulty in uniformly procuring the utmost effect of which the material is capable. Either the common arsenic, if kept dry, and mixed at the time of using, is too coarse to be dissolved by the creosote or other fluid which is used as a vehicle, or it is too crude; or, if it operate well at first, it speedily deteriorates, and soon becomes worthless. There is a good article of arsenic ready prepared with creosote, sold at the dental depots, which works well at first, but which soon looses its quality, and a careful operator will throw the bottle away before a quarter of it is used. It is anything but satisfactory, after making an application of an arsenical preparation, to find that it is unreliable, or that it is deteriorating; that it works effectively in this or that case, but that it can never be depended upon; to find the pulp almost as much alive as it was before the application; that you cannot even open into it, or, if you can, you cannot extirpate any distance. If any man should tell me that he never had any such trouble, I should suspect that he was not in the habit of thoroughly extirpating the pulp, that he devitalized just enough to stop toothache, or to obtund the sensibility to the extent which would enable him to fill the tooth without any immediate periosteal irritation. And by thorough extirpation I do not mean the impracticably exact, unnecessary, and even undesirable following of every narrow and tortuous canal; but I should suspect that he does not even extirpate to the extent which is readily practicable with fine extirpators and broaches, and which is absolutely indispensable for the assurance of success.

It is not sufficient that we have an arsenical preparation which operates satisfactorily at first, or for a while, but which will not retain its properties for any length of time. To say nothing of the expense, or even of the inconvenience of frequent renewals, deterioration must have a period of inception and of gradual increase; and it is unsatisfactory not to know when it has begun, and never to be able to tell when we can command the full power of the material. What we want, is a preparation which will not deteriorate, which will stand on the case a decade, if need be, and out of which the last particle will be as good as the first. Then we shall have something which can be depended upon, and we shall be subjected to no other uncertainties in its use, except those which spring from the constitutional idiosyncrasies of the patient.

I have tried all the different methods of using and combining arsenic, when I thought I would again resort to the dry article, and mix as I used it. In purchasing arsenic from the druggists I have always taken what they gave me, not understanding that there was any difference in the quality. This time, the druggist to whom I applied asked if I wanted the best. Surprised, I answered of course I did; when he gave me some of Squibb's. This preparation is finely ground, white, and almost an impalpable powder. It is a confession of ignorance, or inattentiveness perhaps, but I was unaware of the existence of such an article; and it may contribute to the satisfaction of my fellow practitioners to make them acquainted with it. It is the most efficacious and reliable material I have ever used. I keep it dry, and catch a little of it, about half or quarter the size of a medium sized pin's-head, upon a bit of cotton saturated with creosote, on the point of an excavator, at the time of using. I have had the article on hand now for a year, and I find it works just as well as when I first used it. I do not mean to say that it will destroy the pulp from crown to apex at one application. I should be suspicious of any preparation that was endowed with the strength, or was used in quantity to do so. But it will devitalize the pulp to an extent and with a uniformity which I have never before been able to secure. I can invariably, after allowing the minute portion which I have described to remain not over twenty-four hours, open up the pulp, and remove a large portion of it, and sometimes I am enabled to remove the whole and fill at that sitting; while with other preparations which had been on hand as long as this, I could do little or nothing. With these the nerve might be sufficiently destroyed that it would not ache; but for all purposes of operating it would be as alive as ever. If I used larger quantities I might succeed in destroying the nerve thoroughly at one application, but I should fear that, from a quantity sufficient to destroy the nerve in so short a time, a surplus would be absorbed by the highly vascular cementum, and attack the periosteum in course of time. I prefer the safer method, even though it take longer. I seldom or never make a second application, applying creosote and tanic acid to prevent the decomposition of any portion of the pulp, which, upon filling, I may be unable to remove, and allow the remains of the pulp to slough, which takes about a week. If it is a tooth which shows, I employ but little tanin, as I fear it may add to the discoloration which usually ensues after devitalization, but if it is a back tooth, I use freely, as I find more satisfactory results attending its use than without it. Some operators allow the nerve to slough without attempting to open into it, after removing the application of arsenic; but I am urged to remove all I can, both of dead pulp, decomposed dentine, and of solid bone, by the consideration that the application of arsenic has been absorbed by these parts, and if allowed to remain there a week or so, might do serious injury to the tooth or alveolus. After it has done its first work on the pulp, I want to get rid of it and scrupulously clean and syringe out all that the arsenic has come in contact with, and that I wish to get rid of, until I strike a sensitive part of the nerve. Arsenic is a valuable dental agent in its place, but in bungling or reckless hands great mischief may be done with it; and if the quantity which I have sometimes seen used were allowed to remain in the tooth a week, or even the remains of it after the first application, had been removed, without thorough cleansing, enough could be absorbed to destroy the vitality of a whole jaw. The human economy will stand a great deal of abuse, but that it is not safe to impose upon its powers, the terrible accidents which have been known to attend the inconsiderate use of arsenic attest. It is often the case that in making an application to an irritated nerve, the nerve is so exceedingly sensitive that it is impracticable to remove a mass of decayed dentine which may cover it. When the wax is removed, and the cotton on which the arsenic was applied, no arsenic is visible, although the wax, or other retaining substance, may have been so tight as to preclude the possibility of its all escaping around it. The arsenic may be found, some of it in the dentinal structure, some of it in the pulp, some of it may have escaped around the wax, but most of it is in this spongy mass of decay, awaiting only the presence of moisture and the lapse of time, to penetrate the tooth, or to insensibly assail the periosteum around the margins of the gum, if the cavity is near it. Before the tooth is allowed to stand for the nerve to slough, this mass of decay should be swept away, if nothing more is done, and the arsenic with it. Speaking generally, that cannot be regarded as any other than negligent practice where this is neglected. By this careful method I seldom have any trouble attending the destruction of pulps, and I regard my nerve cases as among the most satisfactory results of my practice. Occasionally I find a little periosteal inflammation and suppuration, but the trouble is slight and transient, and is generally confined to those whose system is depressed by their excesses or other debilitating causes. The mouths of my patients are generally as healthy around a devitalized tooth as around those whose pulps are intact. No unsightly, offensive and troublesome abscesses exist, such as so often proclaim the incompetence, or unfaithfulness of the operator; nor am I mortified by the terrible immediate periosteal inflammation which I have seen attend inconsiderate tampering with the pulp and pulp canal; but clean and healthy gums and alveoli reward a tolerable amount of patience and fidelity, or exemplify the extent to which these virtues may compensate an indifferent ability.

Whilst upon this subject, I would allude to the conviction which has generally obtained, that the necessity of employing arsenious acid for the purpose under consideration has not yet been destroyed by any method of capping by which it has been hoped to save nerves alive. It is due to my friend Dr. Atkinson to say, however, that the employment of oxychloride of zinc, which, as far as I am aware, he was the first to recommend for this purpose, has in my hands come nearer to that object than any other substance I have ever used. I have been surprised and gratified at the results obtained in the mouths of young persons, though for adults it is utterly unreliable.—*Dental Miscellany.*

